# The Structure and Functional Changes of Thyroid in Severe Acute Pancreatitis Rats

**DOI:** 10.33549/physiolres.935403

**Published:** 2025-02-01

**Authors:** Bo YANG, Huanyu QIAO, Yongmin LIU, Xiaona WANG, Wenxing PENG

**Affiliations:** 1 Department of Cardiac Surgery, Beijing Anzhen Hospital, Capital Medical University, Beijing, China; 2 Department of Anesthesia, Beijing Anzhen Hospital, Capital Medical University, Beijing, China; 3 Department of Pharmacy, Beijing Anzhen Hospital, Capital Medical University, Beijing, China

**Keywords:** Severe acute pancreatitis, Thyroid, Structure and functional changes, Transmission electron microscopy

## Abstract

Severe acute pancreatitis (SAP) is associated with metabolic disorders, hypocalcemia, and multiple organ failure. The objective of this study was to investigate changes in thyroid ultrastructure and function in rats with SAP and to provide a theoretical basis for the clinical treatment of thyroid injury in patients with SAP. 64 male SPF Wistar rats were randomly divided into the SAP group and the control group. Pancreatic enzymatic indicators and thyroid hormones were detected, pathology scores were evaluated, and morphological changes were observed under light microscopy and transmission electron microscopy (TEM) in both groups. The serum levels of triiodothyronine (T3), tetraiodothyronine (T4) and Ca^2+^ were significantly lower in the SAP group than in the control group (P<0.05), whereas the level of calcitonin (CT) was significantly higher than that in the control group (P<0.05). The thyroid structure (pathology and electron microscopy) of the SAP rats was seriously damaged and worsened over time. SAP can cause thyroid injury through a variety of mechanisms, which can also retroact to pancreatitis to aggravate the inflammatory response. This study may have theoretical significance for basic research on SAP.

## Introduction

Acute pancreatitis (AP) is a common indication for hospital admission and is often accompanied by systemic manifestations. Most patients with AP develop mild to moderate upper abdominal pain accompanied by vomiting, tachycardia, fever, leukocytosis, and increased pancreatic enzyme levels [1]. This disease is self-limited in the majority of patients and resolves within 1 week [2,3]. Approximately 20 % of patients develop moderate or severe acute pancreatitis (SAP). The incidence of SAP is approximately 20 %, which can be associated with organ failure and complications such as pancreatic necrosis and abscess, leading to septicemia, increased organ failure, and even death [2,3].

The thyroid gland is one of the most important endocrine glands and contains two types of cells: thyroid follicular cells and C cells [4]. The thyroid gland can synthesize a variety of hormones, including triiodothyronine (T3), tetraiodothyronine (T4) and calcitonin (CT), which play significant roles in cellular energy metabolism, substance metabolism, growth and development [4–7]. Recent studies have reported elevated thyroid hormone levels in patients with AP [8,9].

Studies by Peng *et al*. and Fabrès *et al*. showed that AP was often associated with hypocalcemia, which was an independent risk factor for death and served as a potential prognostic factor [10,11]. And the extent of decreased calcium was closely related to thyroid C cells [12,13]. However, changes in thyroid ultrastructure and C cell function in SAP have been reported. Our study aimed to establish a rodent model of SAP injury by retrograde cholangiopancreatography injection of 5 % sodium taurocholate. We observed the functional and morphological changes in the thyroid in SAP and explored the relationship between functional and ultrastructural changes to provide a theoretical basis for the evaluation of pancreatitis.

## Methods

### Animals

64 male SPF Wistar rats (7–8 weeks, 200–250 g, obtained from the Animal Resource Centre of Beijing Anzhen Hospital, Beijing, China) were fasted overnight (12 h, allowed to drink water freely) at room temperature and 12 h day-night rhythm before surgery. All animal procedures were approved by the ethics committee of Beijing Anzhen Hospital, Capital Medical University, and performed in compliance with the Guide for the Care and Use of Laboratory Animals from the National Institutes of Health.

The animals were randomly divided into two groups, including the sham operation group (control group, n=32) and the SAP group (n=32). The SAP model was prepared based on the method of Aho *et al*. [14]. The animals were laparotomized at the midline under intraperitoneal anesthesia with 10 % chloral hydrate (3 ml/kg). No. 4.5 scalp needle was pierced into the main pancreatic duct through the mesangial edge nipple of the duodenum. 5 % Sodium taurocholate (STC) solution (Sigma, USA, dissolved in 0.9 %) 1 ml/kg was injected at a speed of 0.1 ml/min. After clipping the main pancreatic duct for 5 min, pancreatic edema and hemorrhage occurred. In the control group, 0.9 % saline was used instead of STC after anesthesia. After the operation, we closed the abdomen layer by layer and injected saline (20 ml/kg) subcutaneously to compensate for fluid loss during the operation (both groups). The rats in the two groups were further divided into four subgroups at 1, 3, 6 and 12 time points (n=8) after the injection of sodium taurocholate.

### Specimen collection

Blood samples were collected by heart puncture, and pancreatic tissue and thyroid glands were collected at the end of the study. After centrifugation at 2000× g for 10 min, the serum was stored at −80 °C to measure amylase (AMY), lipase (LIPA), phospholipase A2 (sPLA2), T3, T4, CT and Ca^2+^. We dissected the rats and immediately acquired and trimmed all pancreatic tissues and thyroid gland.

### Observations and measurements

AMY, LIPA, sPLA2 and Ca^2+^ were determined by automatic Biochemical Analyzer (Department of Laboratory Medicine, Beijing Anzhen Hospital), T3, T4 and CT were determined by radioimmunoassay (Immunoradiometric assay kit, Chemclin Biotech Co., Ltd, Beijing). In our experiment, the thyroid hormones T3 tested were all total triiodothyronine (TT3), and the T4 tested were all total tetraiodothyronine (TT4).

Specimens were observed under light microscopy and transmission electron microscopy (TEM). After laparotomy, all pancreas and bilateral thyroid tissues were harvested before cardiac arrest. The whole pancreatic tissue and half of the thyroid gland were fixed in 4 % paraformaldehyde and sectioned at 4-μm thick for hematoxylin and eosin (H&E) staining. The left thyroid tissue was observed under TEM (Hitachi H-300, Japan) for ultrastructural morphologic study. This part was pre-fixed with 2.5 % glutaraldehyde, post-fixed with 1 % osmic acid, dehydrated by graded ethanol, embedded (EPON812 Embedding machine, USA), sliced (LKB-V Ultramicrotome, USA) and stained by uranyl acetate – lead citrate.

### Statistical analysis

All data were analyzed using SPSS22.0 software and presented as mean ± standard deviation (SD). Independent samples *t*-test was used to compare differences between two groups. Statistical significance was set at P<0.05.

## Results

### Pancreatitis enzymology

**Fig. 1:**
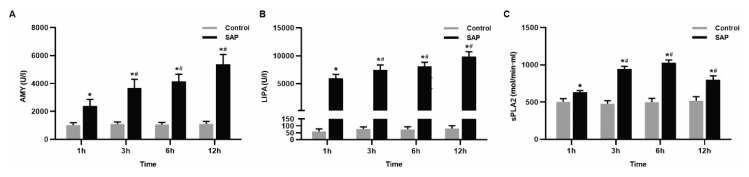
The levels of pancreatitis enzymology. (**A**) The levels of serum amylase activity. The pancreatitis increased the serum AMY activity. (**B**) The levels of serum lipase activity. The pancreatitis increased the serum lipase activity. (**C**) The level of serum sPLA2 was found to increase rapidly at 3 h and reached the peak at 6 h after pancreatitis. Results are expressed as means ± SD of n=8 rats for each subgroup. * P<0.05 vs. control groups, **^#^** P<0.05 vs. SAP 1 h group.

The levels of AMY, LIPA and sPLA2 in the SAP group were significantly higher (P<0.05) than those in the control group. With the extension of time, the three indexes of the SAP group constantly developed and the changes were statistically significant (P<0.05) (Fig. 1).

### Pancreatitis pathology

**Fig. 2:**
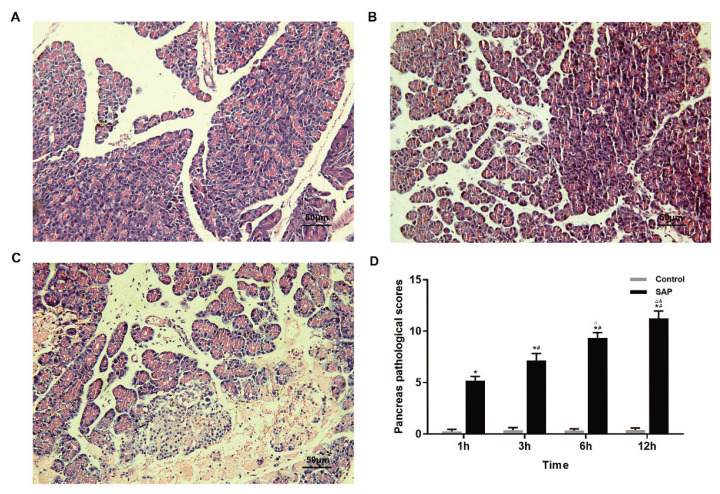
Pancreatic pathology in the light microscopy and the changes of pancreas pathological scores in the SAP and the control groups. (**A**) The pancreatic tissue in control groups, ×200. (**B**) The pancreatic tissue in the SAP 3 h group, ×200. (**C**) The pancreatic tissue in the SAP 12 h group, ×200. (**D**): The pancreas pathological scores of control and SAP groups. Results are expressed as means ± SD of n=8 rats for each subgroup. * P<0.05 vs. control groups, **^#^** P<0.05 vs. the SAP 1 h group, **^△^** P<0.05 vs. the SAP 3 h group, **^&^** P<0.05 vs. the SAP 6 h group.

Under the light microscope, the structure of pancreatic lobules was integral, and the interstitial parts were distinct in the control group. No pathological changes were observed in any of these tissues. In contrast, pancreatic acinar edema, putrescence, bleeding, and increased infiltration of inflammatory cells were observed in the SAP group (P<0.05). And the pathological injury aggravated over time. The pancreas pathological scores [15] of SAP rats at each time point were significantly higher than those of the control group (P<0.05) (Fig. 2).

### Thyroid hormone

**Fig. 3:**
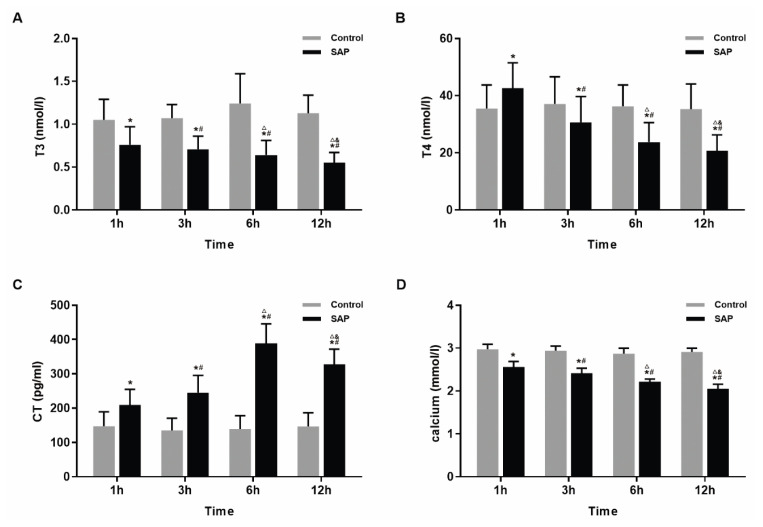
The levels and changes of thyroid hormones and serum calcium. (**A**) The levels of serum T3. The pancreatitis decreased the serum T3 level. (**B**) The levels of serum T4. The level of serum T4 increased at 1 h and fall down from 3 h after pancreatitis. (**C**) The levels of serum CT. CT increased before 6 h and decreased at 12 h after pancreatitis. (**D**) The levels of serum calcium. The calcium continuing declined. * P<0.05 vs. control groups, **^#^** P<0.05 vs. SAP 1 h group, **^△^** P<0.05 vs. SAP 3 h group, **^&^** P<0.05 vs. SAP 6 h group.

Compared with the control group, the level of T3 was significantly lower in the SAP group, and decreased gradually with increasing time points (1 h, 3 h, 6 h and 12 h points) (P<0.05). The T4 level increased at 1 h and decreased after 3 h. These alterations were statistically significant (P<0.05). The CT level increased at each time point in the SAP group (P<0.05), but it showed a decline from 6 h to 12 h. However, it was still higher than that in the control group (P<0.05) within the experimental range. The serum calcium levels in the SAP group were lower than those in the control group (P<0.05) and showed a continuous downward trend over time (Fig. 3).

### Thyroid pathology

**Fig. 4:**
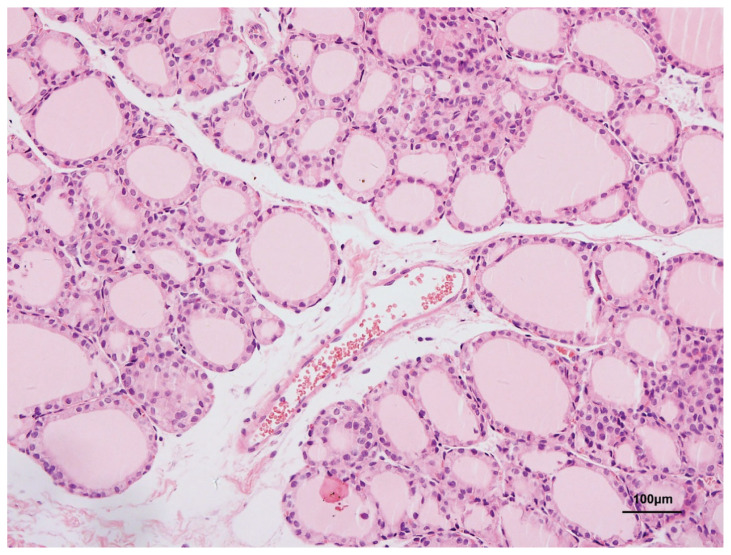
Thyroid tissue in H&E in the control group (×200).

The thyroid follicular cells in the control group were medium-sized and regular. The cavities were filled with colloids. Follicular epithelial cells consisted of simple cuboidal epithelial cells and simple squamous epithelium cells. The interstitial regions were distinct (Fig. 4).

**Fig. 5:**
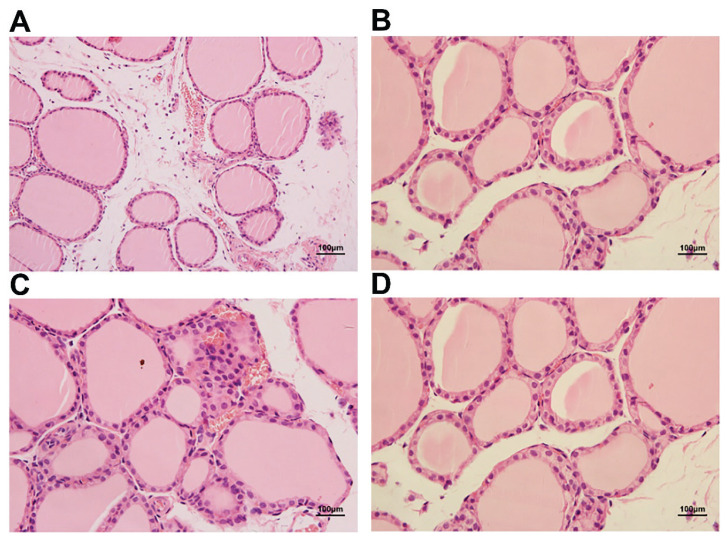
Thyroid tissue in H&E in the SAP group. (**A**) Inflammatory infiltration, thyroid follicular hyperplasia, variety of shape and follicular fusion, ×200. (**B**) Exfoliation of follicular epithelial cells in follicular cavity, ×200. (**C**) Fiber hyperplasia accompanied with capillary congestion, ×400. (**D**) Simple cuboidal epithelium cells and simple squamous epithelium cells were replaced by simple columnar epithelium cells. The colloid stored in follicular cavity was depauperate, ×400.

Inflammatory infiltration, thyroid follicular hyperplasia, morphological changes and follicular fusion were observed in the SAP group (Fig. 5A, B). Simple cuboidal epithelial cells and simple squamous epithelial cells were replaced by simple columnar epithelial cells (Fig. 5C, D). The colloid stored in the follicular cavity was depauperate (Fig. 5D). Follicular epithelial cell exfoliation was observed in the follicular cavity (Fig. 5B). In addition, fiber hyperplasia was accompanied by capillary congestion (Fig. 5C), and bleeding emerged in the thyroid tissue in the early stage (Fig. 5A), followed by hypoperfusion. Moreover, the overall damage was more evident with time.

### Thyroid ultrastructure

**Fig. 6:**
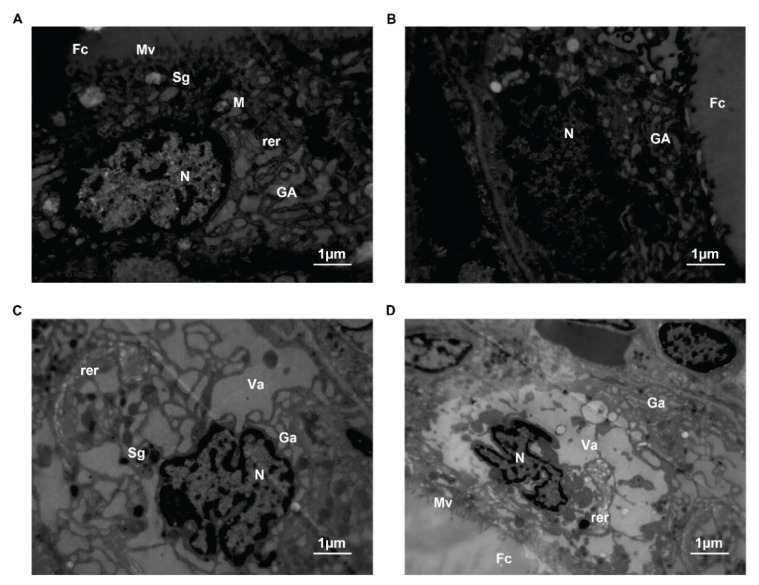
Ultrastructure changes of thyroid follicular cells in transmission electron microscopy. (**A**) Thyroid follicular cells in the control group, ×10000. (**B**) Thyroid follicular cells in the SAP 3 h group, ×10000. (**C**) Thyroid follicular cells in the SAP 6 h group, ×10000. (**D**) Thyroid follicular cells in the SAP 6 h group, ×6000. (N, Nucleus; M, Mitochondria; rer, Rough endoplasmic reticulum; GA, Golgi complex; Sg, Secretory granules; Va, Vacuoles; Fc, Follicular cavity; Mv, Microvilli; CV, Capillary vessel).

In the control group, the membrane of thyroid follicular cells was integrated, and zonula occludens were present. A large number of microvilli were observed on the free surfaces of the cells. Scattered in the cytoplasm were the expansion of rough endoplasmic reticulum, good functional status of mitochondria, Golgi complex, high electron density secretory granules and vesicles of the low electron density colloid. However, in the SAP group, the nucleus presented an irregular form and the nuclear membrane was deeply invaginated. The number of microvilli on the free surface decreased. And intracyto-plasmic organelles showed Golgi complex swelling, endoplasmic reticulum degranulation, mitochondrial vacuolization and secretory granule reduction (Fig. 6).

**Fig. 7:**
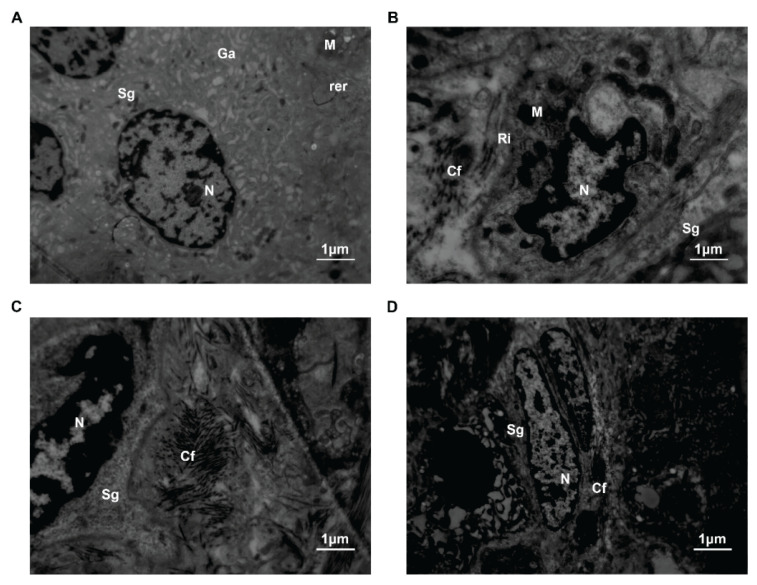
Ultrastructure of thyroid C cells in transmission electron microscopy. (**A**) Thyroid C cells in the control group, ×6000. (**B**) Thyroid C cells in the SAP 3 h group, ×8000. (**C**) Thyroid C cells in the SAP 6 h group, ×12000. (**D**) Thyroid C cells in the SAP 12 h group, ×5000. (N, Nucleus; M, Mitochondria; rer, Rough endoplasmic reticulum; GA, Golgi complex; Sg, Secretory granules; Ri, Ribosomal; Cf, Collagen fibers).

**Fig. 8:**
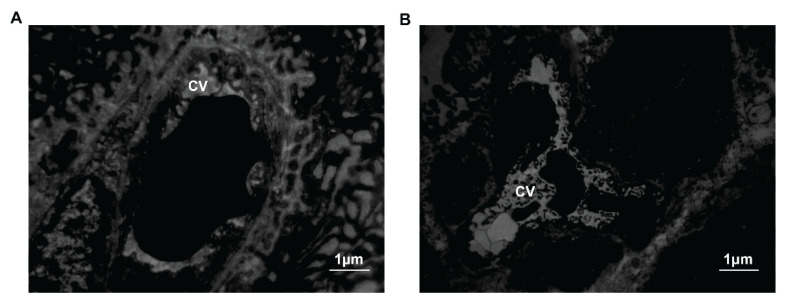
The interstitial parts in the SAP group in transmission electron microscopy. (**A**) The interstitial part in the SAP 3 h group, ×12000. (**B**) The interstitial part in the SAP 6 h group, ×4000 (CV, Capillary vessel).

In the control group, the nuclei of thyroid C cells were round or oval. In the cytoplasm, we discovered secretory granules with low electron density, less fusiform mitochondria, a more developed endoplasmic reticulum and Golgi apparatus and free ribosomes. There was a close connection between C cells and surrounding follicular epithelial cells. In the SAP group, the degree of nuclear membrane invagination increased gradually. The changes in organelles included mitochondrial ribosomal hyperplasia, endoplasmic reticulum and Golgi fusion, and production and secretion of numerous low electron density particles. There was a gap junction between the two cells. At 6 h, the number of organelles began to decrease. Simultaneously, microtubules, microfilaments and neutral fibers proliferated. Cellular organs did not disappear until 12 h. And contact between the cells was not observed anymore (Fig. 7). Early fibrous hyperplasia and capillary congestion could be observed in the interstitium. Then insufficient perfusion was shown (Fig. 8).

## Discussion

Although the mechanism of organ injury, except for the pancreas, in SAP remains unclear, it is currently considered to be related to septicemia and severe traumatic stress [16,17]. The thyroid contains thyroid follicular cells and thyroid C cells, which could aggravate AP and affect the prognosis of AP when injured [18,19]. Thyroid follicular cells can excrete T3 and T4. The results showed that the level of T3 dramatically declined, and the level of T4 raised at 1 h, then fell at 3 h in SAP rats. We also observed changes in cell ultrastructure in SAP rats.

The possible mechanisms are as follows: ① Systemic inflammatory response syndrome (SIRS) commonly occurs in SAP. Then the release of both tumor necrosis factor (TNF-alpha) and vasoactive substances can lead to liver injury [20], resulting in a decrease in the activity of 5′-deiodinase, which catalyzes the conversion of T4 to T3. This process was achieved by competing for the synergistic activation factor of liver type I 5′-deiodinase gene expression and activating the transcriptional regulatory factor NF-κB. The level of T3 decreased. During the early stage of injury, the conversion between T4 and T3 decreases, and T4 accumulates temporarily. Some researchers believe that the body is in a stressed state during pancreatitis [9]. The level of serum cortisol increases under stress, which inhibits the conversion of T4 to T3 [21], resulting in a decrease in T3 and an increase in T4.

In addition, when the immunity of rats was weak and the infection was serious, endotoxin levels in the abdominal cavity would elevate [22]. Endotoxin reached the liver through the portal system, damaging the endomembrane system of Kupffer cells (containing lysosome and epicyte burst) and causing further necrosis of hepatocytes [23,24]. This process reduced the binding rates of thyroid hormone and glucuronic acid. Owing to the decreased levels of conjunction types T3 and T4, which should enter the biliary and enterohepatic circulation under normal conditions, serum T3 and T4 levels decreased [25].

Thyroid hormone synthesis and secretion *in vivo*: Thyroglobulin is a 660 kDa glycoprotein secreted by the thyroid follicular epithelial cells, and its half-life is (29.6±2.8) h. There is a relatively large depot of thyroglobulin in the lumen of the follicles. Arginine binds to thyroglobulin in the rough endoplasmic reticulum and Golgi bodies. After activation by peroxidase, iodine ions in peripheral blood combine with thyroglobulin to form iodinated thyroglobulin, which is then converted to T3 and T4. This process is affected by TSH synthesis *via* adenohypophysis [4]. In this study, the results of transmission electron microscopy showed that follicular epithelial cells showed proliferation and degranulation of rough endoplasmic reticulum, swollen mitochondria, and a decrease of crest. Therefore, oxygen metabolism was weakened and ATP production was reduced. Both expansion of the endoplasmic reticulum and mitochondrial energy metabolism could lead to the disturbance of thyroglobulin synthesis. Simultaneously, the proliferation of connective tissue replaced necrotic follicular epithelial cells. Therefore, the number of functional follicular epithelial cells decreased, which also led to a decrease in the T3 and T4 levels. This phenomenon was also confirmed using light microscopy. The results of H&E staining showed glial atrophy in the thyroid follicular cavity.

When SAP occurs, the body produces a large number of bioactive substances, such as TNF-α, IL-1 and IFN-γ [26]. TNF-α, IL-1 and IFN-γ have been reported to inhibit the synthesis of thyroid hormones by inhibiting cAMP production in thyroid follicular cells [27]. Furthermore, TNF-α could inhibit the follicular epithelial cells on the intussusception of iodine ions. IL-1 affected on the combination of thyroid-binding globulin and T4. IFN-γ could inhibit the expression of peroxidase genes. All of these factors could affect the synthesis and secretion of thyroid hormones.

Thyroid C cells secreting calcitonin originate from the neural crest [28]. C cells can absorb amine precursors, promote decarboxylation, produce procalcitonin, secrete bioactive calcitonin and maintain calcium balance [4,29].

Currently, the mechanism of hypocalcemia in patients with SAP is not yet entirely clear. There are five possible theories: ① saponification theory; ② hypoalbuminemia theory; ③ internal environment hormonal regulation theory; ④ free fatty acid theory; ⑤ changes in cell membrane permeability [27]. This study investigated the structure and function of thyroid C cells and provided evidence of the mechanism of hypocalcemia in pancreatitis.

Our results showed that the serum calcitonin levels in the SAP group were higher than the control group. Transmission electron microscopy revealed that mitochondria and free ribosomes in the cytoplasm proliferated. The rough endoplasmic reticulums and Golgi bodies were fused. Large numbers of particles with low electron density were produced and secreted. What was the reason for these phenomena?

Thyroid C cells contain 1,25-(OH)_3_D receptors. Free vitamin D in the peripheral bloodstream acts directly on C cells and increases the secretion of calcitonin [30,31]. A previous study found that serum vitamin D binding protein levels were significantly decreased in SAP [32]. Because of the reduction of vitamin D carriers, the level of free-form vitamin D increases, and it acts as 1,25-(OH)_3_D receptor on C cell surface, which increases the secretion of calcitonin. Another study found that gastrin and glucagon secretion increased significantly in the early stage of AP [33]. Gastrin and glucagon regulate calcitonin secretion. Therefore, we further speculated that the body can also promote calcitonin secretion by increasing gastrin and glucagon levels in SAP. In addition, a large number of cytokines such as IL-1 and NF-κB are released into the blood stream. Studies have shown that these cytokines could increase the transcription of calcium-sensing receptor gene (CASR gene) [34]. CASR gene opens non-specific ion channels by activating phospholipase C. Calcium influx leads to membrane depolarization and L-type calcium ion channels open to stimulate the secretion of calcitonin. CASR gene inhibits parathyroid cell proliferation and parathyroid hormone gene expression [35–37]. Therefore, it can lead to a decrease in parathyroid hormone levels and progression of hypocalcemia.

We found that serum calcitonin levels decreased after 6 h. Possible reasons could be considered as follows: ① Gastrin and glucagon only increased in the early stage of AP, and the promotion of calcitonin weakened from 6 h; ② As shown by electron microscopy, the body developed microcirculation hypoperfusion at about 6 h. The hormone secretion of C cells was not dominated by the nervous system, but was related to the microenvironment. The sensitivity of C cells to plasma cytokines and hormones decreased during insufficient perfusion. ③ Apoptosis of the cells and reduction of organelles after 6 h also affected hormone synthesis and secretion. This consequence above can be confirmed by the results of electron microscopy.

## Conclusions

In our study, the structure and function of the thyroid gland were found to be impaired in SAP. These changes can also cause pancreatitis and exacerbate the inflammatory response. However, the protection of the tissue from injury was not involved in this study. However, this requires further research.
